# Large variation of magnetic properties of amorphous Fe–Zr thin films with Ar pressure during sputtering

**DOI:** 10.1038/srep41894

**Published:** 2017-01-31

**Authors:** Miri Kim, Nark-Eon Sung, Sang Ho Lim

**Affiliations:** 1 Department of Nano Semiconductor Engineering, Korea University, Seoul 02841, Korea; 2 Energy and Environmental Research Team, Beamline Division, Pohang Accelerator Laboratory, Pohang 37673, Korea; 3 Department of Materials Science and Engineering, Korea University, Seoul 02841, Korea

## Abstract

A large change is observed in the magnetic properties of amorphous Fe–Zr thin films sputtered at different Ar pressures. The change depends on the composition of the alloys and at compositions near 60 at.% Fe, for example, the magnetisation measured at 10 kOe increases 30-fold with an increase in the Ar pressure from 2 to 10 mTorr. The magnetic properties are well explained by a combination of two phenomena—superparamagnetism and spin glass behaviours—and the large change is partly related to the number density of a magnetically correlated region. Examinations of the microstructure by X-ray diffraction, transmission electron microscopy, and X-ray absorption fine structure spectroscopy reveal no appreciable difference in it as a function of the Ar pressure. This indicates that even a very slight change in the microstructure can greatly affect the magnetic properties of amorphous Fe–Zr thin films, thereby opening up the possibility of employing the magnetic properties of amorphous alloys for the characterisation of amorphous microstructures.

The magnetic properties of amorphous or nanocrystalline materials are highly sensitive to their microstructure. Chudnovsky showed that the permeability and coercivity of amorphous magnetic materials vary sensitively with the structural correlation length^1^. Herzer^2^ reported a similar behaviour in nanocrystalline magnetic materials, revealing that the coercivity varies with D^6^ (where D is the grain size) whereas the permeability varies with D^−6^. Fe–Zr alloys can be suitable candidates for examining the relationship between their magnetic and microstructural properties, because an amorphous phase can be formed over a wide composition range, although the specific composition range depends on the fabrication method^3^,^4^,^5^,^6^,^7^,^8^,^9^,^10^,^11^. Melt spinning has been used to form an amorphous phase over a limited composition range, near the Fe-rich eutectic composition of 90 at.% Fe^3^,^4^,^5^,^6^. Mechanical alloying permits formation of an amorphous phase over a wide composition range of 30–80 at.% Fe^7^,^8^, which is located between the two eutectic compositions of 25 and 90 at.% Fe^12^. Given that mechanical alloying is based on a solid-state reaction that usually begins with pure elemental powders, a long time is required to form an amorphous phase; therefore, the materials in this method are subjected to contamination during the process^7^,^8^. Given its rapid and simple process and its ability to form an amorphous phase over a very wide composition range, sputtering offers distinct advantages for the formation of an amorphous phase in comparison to the two above-mentioned methods^9^,^10^,^11^.

For sputtered thin films with an ‘apparent’ amorphous structure, the degree of amorphization differs depending on the sputtering condition. Among the various sputtering conditions, the Ar pressure during sputtering (*P*_Ar_) is one of the most efficient factors for controlling the degree of amorphization, since it directly affects the thermalization process. Atoms sputtered from the target as a consequence of momentum transfer from Ar ions undergo scattering with Ar atoms or ions on their way to the substrate. This thermalization process, which causes the sputtered atoms to lose their momentum, will be stronger at a higher *P*_Ar_ value. Therefore, the sputtered atoms arriving at the substrate will be less energetic, thus resulting in a higher degree of amorphization. Some scattered results have been reported that demonstrate a large change in the magnetic properties as a function of *P*_Ar_, such as the magnetocaloric effects in Er–Co thin films^13^ and the coercivity and magnetostriction in Tb–Fe thin films^14^. In order to understand the relationship between the degree of amorphization and the magnetic properties more clearly, a systematic study was conducted that involved fabrication of amorphous Fe–Zr alloy thin films over a wide composition range and characterisation of these films both structurally and magnetically. Recently, thin films of amorphous Fe–Zr alloys have found important applications in the control of the texture and surface roughness as a way to improve the related magnetic and magnetotransport properties^15^,^16^. Despite this practical importance of these alloys, their magnetic properties are not clearly understood at present. A clear understanding of the relationship between the degree of amorphization and the magnetic properties of amorphous Fe–Zr alloys will therefore be of great theoretical and practical importance.

## Results

### Variation of magnetic properties with *P*_Ar_

**Figure 1:**
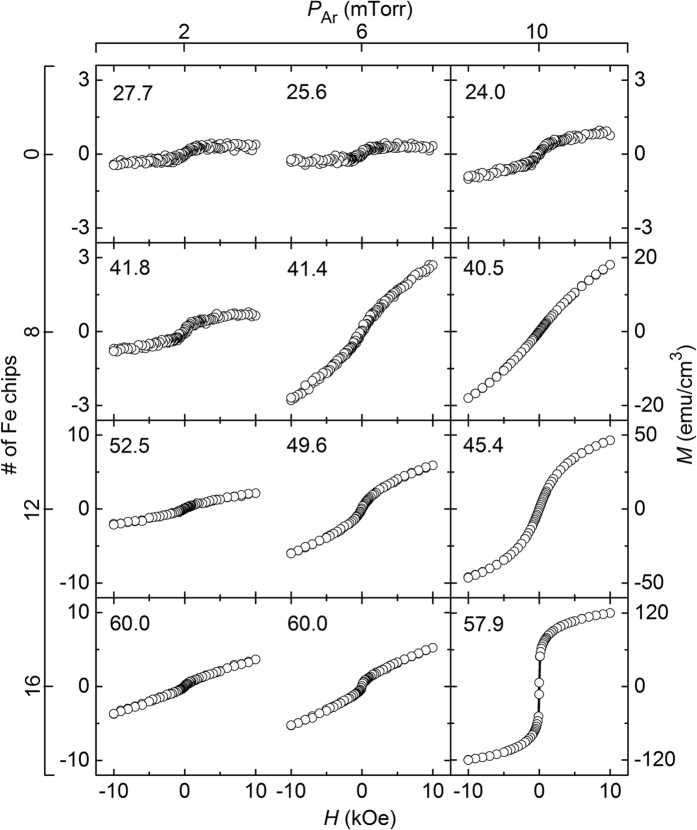
*M*–*H* loops measured at room temperature for all samples fabricated in this study, as functions of *P*_Ar_ (horizontal axis) and number of Fe chips on alloy target (vertical axis). The numeral in each loop denotes the *C*_Fe_ value. Note the difference in the scale of the *y*-axis (*M*) for the samples fabricated at *P*_Ar_ = 10 mTorr when the number of chips is 8, 12, and 16.

**Figure 2:**
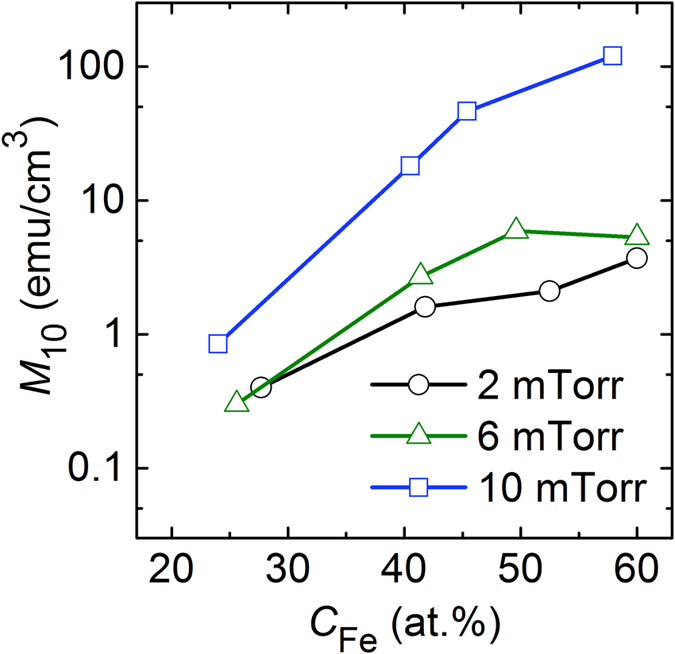
Results of *M*_10_ as a function of *C*_Fe_ at three different *P*_Ar_ values: 2 (circles), 6 (triangles), and 10 mTorr (squares). Note that the *y*-axis for *M*_10_ is expressed in the logarithmic scale, whereas the *x*-axis for *C*_Fe_ is expressed in the linear scale.

Figure 1 shows *M*–*H* (where *M* and *H* are the magnetisation and applied magnetic field, respectively) loops measured at room temperature for all the samples fabricated in this study, as functions of *P*_Ar_ (horizontal axis) and of the number of Fe chips on the Fe_20_Zr_80_ (at.%) alloy target (vertical axis). Here, the samples are denoted in parentheses by two numerals separated by a comma; the first numeral represents the Fe content in at.% (*C*_Fe_) and the second one represents the *P*_Ar_ value in mTorr during sputtering. *C*_Fe_ and *P*_Ar_ are the two most important parameters influencing the magnetic properties. For a given number of Fe chips, the *C*_Fe_ value decreases with increasing *P*_Ar_, because Fe is lighter than Zr and hence subjected to a greater degree of thermalization at a higher *P*_Ar_ value. At a given composite-target configuration, however, the difference in *C*_Fe_ is not large; this difference is within several percent in the *P*_Ar_ range investigated in this study, as can be understood from the *C*_Fe_ values shown in Fig. 1. A prominent point to be noted from the results shown in Fig. 1 is that the magnetisation is highly sensitive to *P*_Ar_; this is particularly true when the *C*_Fe_ value (or the number of Fe chips) is large. The increase in *M* with an increase in *P*_Ar_ from 2 to 6 mTorr is rather small, but in most cases, the increase in *M* with an increase in *P*_Ar_ from 6 to 10 mTorr is very large. Note that for a nonzero number of Fe chips, the scale for *M* at *P*_Ar_ = 2 and 6 mTorr is different from that at *P*_Ar_ = 10 mTorr. For the samples fabricated using the target with 16 Fe chips, in which case the *C*_Fe_ value is near 60 at.%, a large change in magnetisation occurs as a function of *P*_Ar_. At *P*_Ar_ = 2 mTorr, the *M* value at 10 kOe (*M*_10_) is 3.7 emu/cm^3^; this is similar to the value observed for the samples with smaller *C*_Fe_; however, it increases considerably to 120 emu/cm^3^ at *P*_Ar_ = 10 mTorr. It should be noted that the large change in *M* occurs as a function of *P*_Ar_ only, because for a given composite-target configuration, the *C*_Fe_ value also varies with *P*_Ar_, though the variation is small. It is therefore necessary to view the change in *M* as a function of both *P*_Ar_ and *C*_Fe_. Figure 2 shows the results of *M*_10_ as a function of *C*_Fe_ at three different *P*_Ar_ values: 2 (circles), 6 (triangles), and 10 mTorr (squares). Note that the *y*-axis for *M*_10_ is expressed in the logarithmic scale whereas the *x*-axis for *C*_Fe_ is expressed in the linear scale. At a given *C*_Fe_ value, the *M*_10_ values at *P*_Ar_ = 10 mTorr are significantly higher than those at *P*_Ar_ = 2 and 6 mTorr, which is particularly true for the samples with *C*_Fe_ > 40. These results indicate that the much larger *M* values of the samples fabricated at *P*_Ar_ = 10 mTorr than those of the samples fabricated at lower *P*_Ar_ values are due mainly to *P*_Ar_, and not *C*_Fe_. It will be of interest to locate a more precise *P*_Ar_ value showing a large jump in *M*. To this end, additional experiments were performed at two different *P*_Ar_ values of 4 and 8 mTorr using the target with 16 Fe chips, in which case the *C*_Fe_ value is near 60 at.%. The critical *P*_Ar_ value should be located between 6 and 8 mTorr, as the sample fabricated at *P*_Ar_ = 8 mTorr exhibits a high *M*_10_ value of 74.9 emu/cm^3^, which is of comparable magnitude observed for the sample fabricated at *P*_Ar_ = 10 mTorr (120 emu/cm^3^).

**Figure 3:**
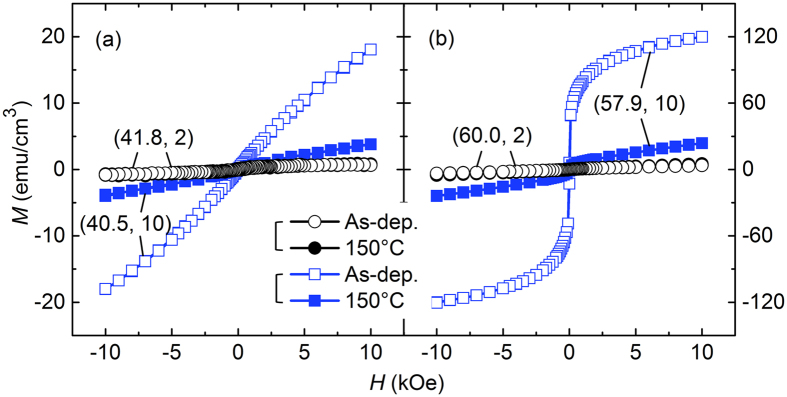
*M*–*H* loops of samples fabricated from two different composite-target configurations with (**a**) 8 and (**b**) 16 Fe chips at two different *P*_Ar_ values of 2 (circles) and 10 mTorr (squares) in as-deposited state (open symbols) and after annealing at 150 °C (closed symbols). The first and second numerals in parentheses separated by a comma denote the *C*_Fe_ and *P*_Ar_ values, respectively.

In consideration of the relationship between *P*_Ar_ and the degree of amorphization, the results shown in Figs 1 and 2 can be interpreted as a large *M* value at a high degree of amorphization. In order to confirm this interpretation, some samples were annealed at a low temperature of 150 °C, where only a structural relaxation is expected to occur. Indeed, a large change in the magnetic properties was observed after the low-temperature annealing; as an example, results for the samples fabricated from two different composite-target configurations with 8 (*C*_Fe_ ~ 40) and 16 (*C*_Fe_ ~ 60) Fe chips are shown in Fig. 3(a) and (b), respectively. For the samples fabricated at 2 mTorr, at which pressure the formation of a more relaxed amorphous phase is expected, only a minor change in *M* occurs after the annealing (the two sets of results in Fig. 3(a) and (b) nearly overlap). This is not the case with the samples fabricated at 10 mTorr, however; the magnetisation at this *P*_Ar_ value reduces greatly after the annealing in such a way that the *M* value of the annealed samples is of a level similar to that of the samples fabricated at *P*_Ar_ = 2 mTorr. Specifically, for the (40.5, 10) sample, the *M*_10_ value is 18.1 emu/cm^3^ in the as-deposited state, but after the low-temperature annealing, it reduces to 3.86 emu/cm^3^. A similar behaviour is observed for the (57.9 10) sample, whose *M*_10_ value reduces from 120 to 23.8 emu/cm^3^ upon annealing.

### Phenomenological explanation of magnetic properties based on superparamagnetism and spin glass behaviours

For all the samples investigated in this study, no hysteresis is observed in the loops, which is indicated by both zero remanence and zero coercivity. Another noteworthy feature is that the magnetisation does not saturate even at a high magnetic field of 10 kOe, except in some samples with low *C*_Fe_ values. It is predicted that the former is related to the superparamagnetic behaviour^17^, whereas the latter is due to the spin glass behaviour, which is often observed in Fe-based amorphous alloys where the Fe–Fe distance is widely distributed and which results in both ferromagnetic and antiferromagnetic coupling between Fe atoms^18^. On the basis of this prediction, the following equation for *M* consisting of two terms describing the superparamagnetic (Langevin equation) and spin glass behaviours was used to analyse the magnetisation behaviour:


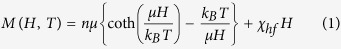


**Figure 4:**
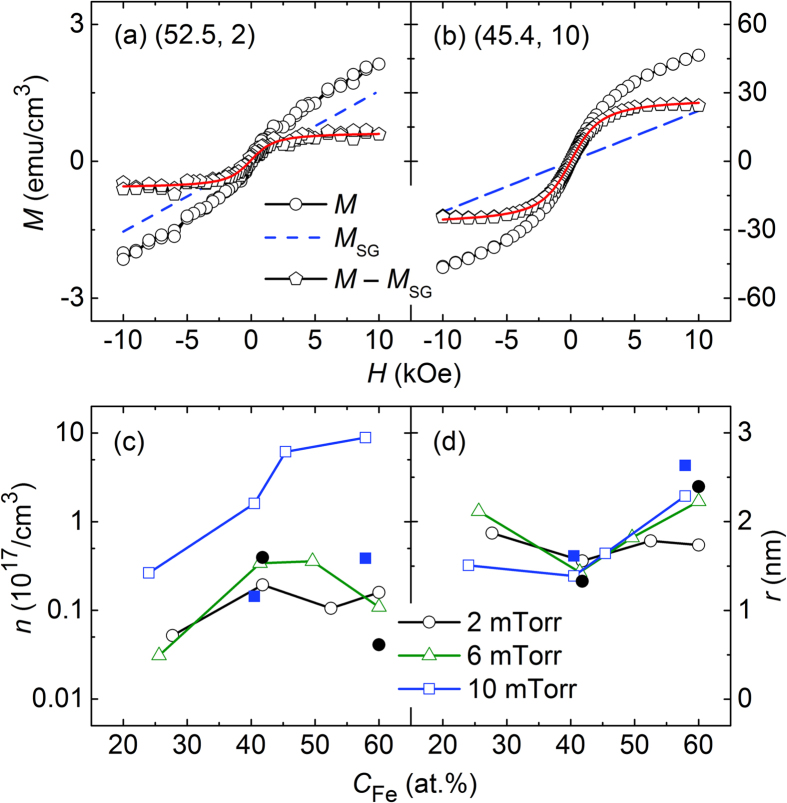
Results of *M* (circles), *M*_SG_ (broken line), and *M* − *M*_SG_ (pentagons) as functions of *H* for (**a**) (52.5, 2) and (**b**) (45.4, 10) samples. Langevin fitting results (solid line) are also shown in relation to the results of *M* − *M*_SG_. Results of (**c**) *n* and (**d**) *r*, which are extracted from Langevin fitting, as functions of *C*_Fe_ at three different *P*_Ar_ values of 2 (circles), 6 (triangles), and 10 mTorr (squares) in as-deposited state (open symbols) and after annealing at 150 °C (closed symbols).

Here, *n* and *μ*, which are two important parameters of the Langevin equation, denote the number density of superparamagnetic particles and their magnetic moment, respectively^17^. Further, *k*_B_ is the Boltzmann constant, *T* is the absolute temperature, and *χ*_hf_ is the high-field magnetic susceptibility. With the accurate values of *χ*_hf_ obtained from the slope of the magnetisation curve in the high-*H* region, the magnetisation contribution of spin glass (*M*_SG_) can be calculated from the second term of Eq. (1). Then, only two fitting parameters, *n* and *μ*, remain in the Langevin equation. Most of the magnetisation curves are well-fitted with Eq. (1), and examples of results are shown in Fig. 4(a) and (b) for two different samples, (52.5, 2) and (45.4, 10), respectively. Indeed, the contribution of the superparamagnetic behaviour (*M*_SPM_, determined by the difference *M*–*M*_SG_) is well-fitted with the Langevin equation over the entire *H* range (solid line), demonstrating that the magnetisation behaviour can be explained by a combination of two phenomena: superparamagnetism and spin glass behaviour. At a given composite-target configuration, the *M*_SG_ value tends to increase with increasing *P*_Ar_. For samples with *C*_Fe_ ~ 40, the *M*_SG_ value at *P*_Ar_ = 2 mTorr is 0 with the occurrence of saturation of magnetisation at low *H* values, but it is nonzero at *P*_Ar_ = 6 and 10 mTorr (with the *M*_SG_ values at 10 kOe being 1.83 and 14.6 emu/cm^3^, respectively). A similar behaviour is also observed for samples with higher *C*_Fe_ values near 50; in this case, the *M*_SG_ value at 10 kOe is nonzero (1.54 emu/cm^3^) at *P*_Ar_ = 2 mTorr and it increases significantly to 22.1 emu/cm^3^ at *P*_Ar_ = 10 mTorr. These results indicate that the spin glass behaviour tends to be stronger at a higher *P*_Ar_ value, and this tendency is stronger at a higher *C*_Fe_ value. In addition to the *M*_SG_ value, it is of interest to examine its relative value, such as *M*_SG_/*M*_10_. For the samples fabricated at *P*_Ar_ = 2 mTorr, the *M*_SG_/*M*_10_ values are 0, 0.73, and 0.75 at *C*_Fe_ = 41.8, 52.5, and 60.0 respectively, indicating a stronger spin glass (or weaker superparamagnetic) behaviour at a higher *C*_Fe_ value. The opposite behaviour is observed for the samples fabricated at *P*_Ar_ = 10 mTorr, where the *M*_SG_/*M*_10_ values are 0.81, 0.48, and 0.17 at *C*_Fe_ = 40.5, 45.4, and 57.9, respectively.

Of the two parameters *n* and *μ*, it would be more convenient to convert the latter parameter into the size of a superparamagnetic particle, such as its radius (*r*). The conversion, however, requires two simplifying assumptions: the particle has a spherical shape and the magnetic moments are due only to Fe atoms, where their magnetic moments are identical to that of bulk Fe (2.2 μ_B_/Fe atom). Figure 4(c) and (d) show *n* and *r* as a function of *C*_Fe_ at three different *P*_Ar_ values: 2 (circles), 6 (triangles), and 10 mTorr (squares). Considering that the *y*-axis for *n* is expressed in the logarithmic scale but that for *r* is expressed in the linear scale, the variation of *n* is extremely large but that of *r* is small. The values of the latter are distributed in the range of 1.5–2.5 nm; furthermore, no notable trend, except for a slight dip near *C*_Fe_ ~ 40, is observed. For the samples fabricated at *P*_Ar_ = 2 and 6 mTorr, the difference in *n* is rather small and a broad maximum occurs at intermediate *C*_Fe_ values. However, for the samples fabricated at *P*_Ar_ = 10 mTorr, the *n* value increases significantly with *C*_Fe_; at the *C*_Fe_ values examined in this study, the *n* value increases significantly from 1.6 × 10^16^/cm^3^ to 8.9 × 10^17^/cm^3^. A comparison of the results of *n* in Fig. 4(c) with those of *M*_10_ in Fig. 2 reveals a similar trend, particularly at *P*_Ar_ = 10 mTorr; this indicates that the parameter *n* is a major factor affecting the magnetisation of the samples used in this study. This consideration is further supported by the change in *n* and *r* upon annealing, the results of which are also shown in Fig. 4(c) and (d) and are indicated by closed symbols. Again, the change in *r* upon annealing is small, without any definite trend with respect to *P*_Ar_ and *C*_Fe_. This also applies to *n* for the samples fabricated at *P*_Ar_ = 2 mTorr. However, for the samples fabricated at *P*_Ar_ = 10 mTorr, a massive reduction in *n* occurs upon annealing, which explains a similar change that occurs in the magnetisation. Specifically, for the samples with *C*_Fe_ = 40.5 and 57.9, the *n* values are 1.6 × 10^17^/cm^3^ and 8.9 × 10^17^/cm^3^, respectively, in the as-deposited state; however, upon annealing, these values decrease significantly to 1.4 × 10^16^/cm^3^ and 3.9 × 10^16^/cm^3^, respectively. For the same samples, the *M*_10_ values are 18.1 emu/cm^3^ and 120 emu/cm^3^ in the as-deposited state; however, upon annealing, these values decrease to 5.1 emu/cm^3^ and 23.8 emu/cm^3^, respectively. These annealing effects further demonstrate the dominant role of *n* in the magnetisation.

### Microstructural characterisation by X-ray diffraction (XRD)

**Figure 5:**
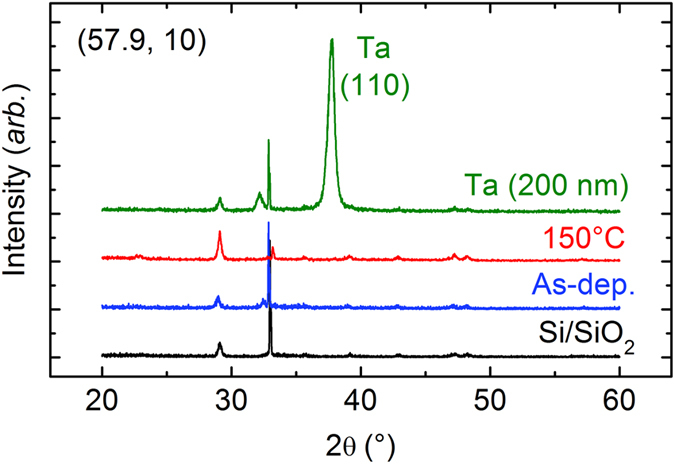
XRD patterns of (57.9, 10) sample in as-deposited state and after annealing at 150 °C. The XRD results for the substrate (Si/SiO_2_) only and the substrate with a thick (200 nm) Ta capping layer (Si/SiO_2_/Ta (200 nm)) are also shown.

The microstructures of all the samples (with a stack structure of Si/SiO_2_ (substrate)/Fe–Zr (200 nm)/Ta (5 nm)) investigated in this study were examined by XRD; Fig. 5 shows the XRD results for the (57.9, 10) sample in the as-deposited state and also after annealing at 150 °C. For comparison, the XRD results for the substrate only and for the substrate with a thick (200 nm) Ta capping layer (Si/SiO_2_/Ta (200 nm)) are also shown. No crystalline peaks resulting from the main layer of the Fe–Zr alloys are observed for the as-deposited and annealed samples, indicating that the microstructure consists of an amorphous phase. All the observed XRD peaks in Fig. 5 are attributed to the substrate and the Ta capping layer. No obvious difference is observed in the XRD results for different values of the experimental parameters *P*_Ar_ and *C*_Fe_ and after annealing.

### Microstructural characterisation by high-resolution transmission electron microscopy (HR-TEM)

**Figure 6:**
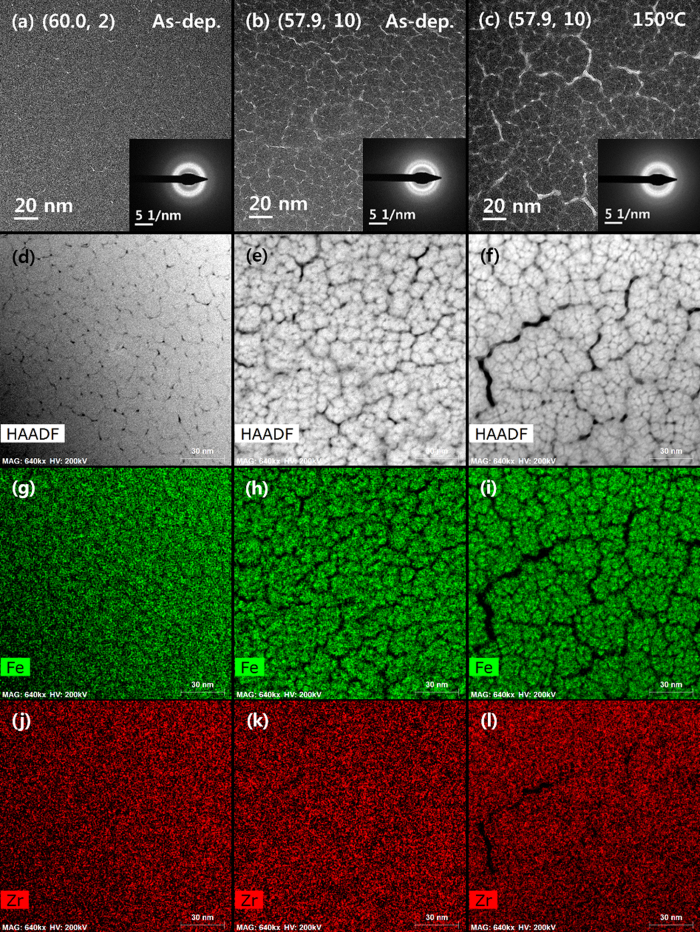
Plan-view TEM images of as-deposited (**a**) (60.0, 2) and (**b**) (57.9, 10) samples and of (**c**) annealed (57.9, 10) sample, together with SAED patterns shown in the insets. (**d**–**f**) HAADF images, (**g**–**i**) elemental mapping results for Fe, and (**j**–**l**) those for Zr, all for the same set of samples.

In order to characterise the microstructure further, the samples with *C*_Fe_ ~ 60 were examined by HR-TEM; these results are shown in Fig. 6(a)–(l). Plan-view images obtained from the two as-deposited (60.0, 2) and (57.9, 10) samples, shown in Fig. 6(a) and (b), respectively, appear to differ substantially as the microstructure of the former is more homogeneous than that of the latter. In principle, however, the microstructures of both samples consist of a similar columnar structure, which is frequently observed in sputtered thin films; nevertheless, this feature is clearer in the (57.9, 10) sample. According to Thornton’s zone model^19^, a columnar structure with clear open boundaries, such as that shown in Fig. 6(b) for the as-deposited (57.9, 10) sample, usually forms at high *P*_Ar_ values, at which the adatom diffusion is insufficient to overcome the shadowing effects of the initial nucleation. At low *P*_Ar_ values, however, the adatoms are still energetic owing to small scattering, and therefore, the adatom diffusion is sufficient to overcome the initial nucleation or the roughness of the substrate; this results in an almost homogeneous texture with few open boundaries, such as that observed in Fig. 6(a) for the as-deposited (60.0, 2) sample. Two diffuse rings without any bright spots, which are observed in the selected area electron diffraction (SAED) patterns shown in the insets of Fig. 6(a) and (b), indicate the formation of an amorphous phase in both samples.

The annealed (57.9, 10) sample was also examined by TEM; its plan-view image is shown in Fig. 6(c). It is recalled that the magnetic properties of the annealed (57.9, 10) sample are similar to those of the (60.0, 2) sample (both in the as-deposited and the annealed states) (refer to Fig. 3(b)). These two samples are therefore expected to exhibit similar microstructures. This expectation, however, is far from met, as the annealed (57.9, 10) sample exhibits an even more pronounced columnar structure with more clearly defined open boundaries (Fig. 6(c)). Here, macro-columns with clearer boundaries consist of several micro-columns; a closer examination of the TEM image of the as-deposited (57.9, 10) sample also shows this feature, albeit with a weaker tendency. The SAED pattern of the annealed (57.9, 10) sample, depicted in the inset of Fig. 6(c), also shows two diffuse rings, indicating that an amorphous phase is maintained upon annealing, in accord with the XRD results. In contrast to the TEM images, the SAED patterns of the as-deposited (60.0, 2) and annealed (57.9, 10) samples—which exhibit similar magnetic properties—have greater similarity than do those of the as-deposited (60.0, 2) and (57.9, 10) samples. This may provide a clue to explaining the difference in the magnetic properties, although at present, it is difficult to relate the shape of the SAED pattern to the degree of amorphization (and the resultant magnetic properties).

The columnar structure is visible more clearly in high-angle annular dark-field (HAADF) images acquired by scanning TEM (STEM), which are shown in Fig. 6(d)–(f). The elemental distributions of Fe and Zr were examined by energy dispersive X-ray spectroscopy (EDS), and the results are shown in Fig. 6(g)–(i) for Fe and in Fig. 6(j)–(l) for Zr. The distribution of Fe, which is less uniform than that of Zr, has a similar shape to the columnar structure, indicating that the distribution of Fe atoms is mainly responsible for the columnar structure. In the case of the as-deposited (60.0, 2) sample, this feature is clearer when the mapping results are obtained at a higher resolution (see Supplementary Fig. S1). Although the TEM images and EDS elemental mapping results provide more valuable microstructural information than do the XRD results, the former two approaches are still insufficient in explaining the large change in the magnetic properties as a function of *P*_Ar_.

### Microstructural characterisation by X-ray absorption fine structure (XAFS)

**Figure 7:**
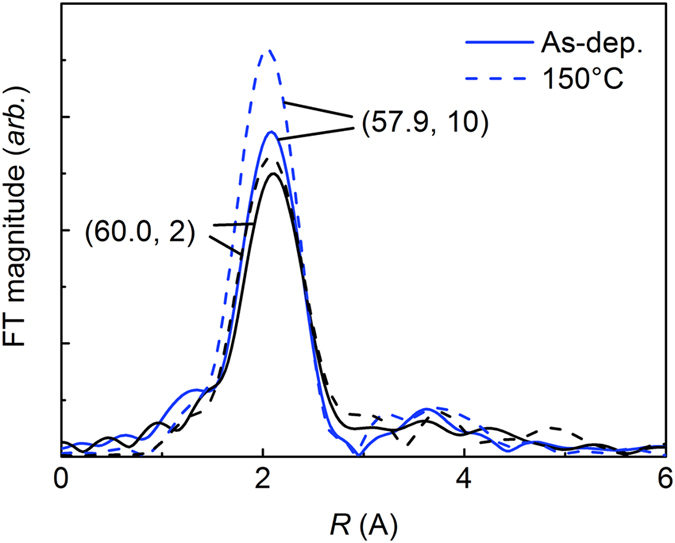
Fourier-transformed EXAFS spectra at Fe-K edge for (60.0, 2) and (57.9, 10) samples in as-deposited state (solid lines) and after low-temperature annealing (dashed lines).

In an effort to obtain information on the degree of amorphization, XAFS experiments were performed, from which the energy dependence of the X-ray absorption coefficient at and above the absorption edge of a selected element could be measured in two different modes: the transmission and fluorescence modes^20^. The former mode offers an important advantage of directly measuring the absorption coefficient from incident and transmitted beam intensities and hence obtaining reliable information. However, given the nature of the transmission, this technique is unsuitable for a thin-film sample with a thick substrate, as is the case in this study. Even though samples with varying geometries can be measured in the fluorescence mode, the quality of the measured results is generally poorer than that in the transmission mode, particularly in the high-energy range. Despite this, some valuable information was obtained in the fluorescence mode. X-ray absorption near-edge structure (XANES) results measured at the Fe-K edge indicate that Fe atoms are in the pure metallic state even after the low-temperature annealing (see Supplementary Fig. S2). Fourier-transformed extended XAFS (EXAFS) spectra are shown in Fig. 7 for the (60.0, 2) and (57.9, 10) samples in the as-deposited state (solid lines) and after the low-temperature annealing (dashed lines). All the spectra are theoretically fitted with a bcc Fe model to extract related parameters. The *R* factor, which quantifies the discrepancy between measured and theoretically fitted results, is very low—in the range of 0.003–0.014; this indicates that the theoretical fitting with a bcc Fe model is reliable. The distance to the first nearest neighbour Fe atoms (*R*_Fe-Fe_) is in the range of 2.445–2.477 Å, which is similar to the nearest neighbour Fe-Fe distance (2.473 Å) in bcc Fe. Two main factors affect the magnitude of Fourier-transformed EXAFS spectra: the Debye–Waller factor (σ^2^), which describes the mean-square relative displacement of *R*_Fe-Fe_, and the number of Fe atoms at the distance of *R*_Fe-Fe_ (*N*_Fe-Fe_). The σ^2^ values are nearly the same for all the samples, in the range of 0.011–0.012, indicating that *N*_Fe-Fe_ is the main factor affecting the magnitude of Fourier-transformed EXAFS spectra. The *N*_Fe-Fe_ values are 3.00 ± 0.55 and 3.30 ± 0.83 for the as-deposited (60.0, 2) and (57.9, 10) samples, respectively, and these values increase to 3.09 ± 0.83 and 4.24 ± 0.69 upon annealing. The change in *N*_Fe-Fe_ (and hence in the magnitude of Fourier-transformed EXAFS spectra) upon annealing is significantly larger for the (57.9, 10) sample than for the (60.0, 2) sample. This can serve as evidence for the result that the (57.9, 10) sample in the as-deposited state is less relaxed than the (60.0, 2) sample. However, XAFS results such as those of *N*_Fe-Fe_ and their change upon annealing are unable to quantify the difference in the degrees of amorphization and hence unable to explain the large change in the magnetic properties with a change in *P*_Ar_.

## Discussion

Given that no obvious crystalline peaks were detected in the XRD patterns (Fig. 5), the Fe–Zr alloy thin films examined in this study consist of an amorphous phase. This is further confirmed by two diffuse rings observed in the SAED patterns (insets of Fig. 6(a)–(c)). Despite this microstructural feature of a single amorphous phase, the magnetic properties undergo a large change as a function of *P*_Ar_ during sputtering; for example, the *M*_10_ value of the samples with *C*_Fe_ ~ 60 is 3.7 emu/cm^3^ at *P*_Ar_ = 2 mTorr, but it is as high as 120 emu/cm^3^ at *P*_Ar_ = 10 mTorr. A phenomenological analysis of the magnetic properties indicates a combination of superparamagnetism and spin glass behaviours. For the samples fabricated at *P*_Ar_ = 10 mTorr, which exhibit *M* values much larger than those of samples fabricated at low *P*_Ar_ values, the superparamagnetic behaviour becomes stronger at a higher *C*_Fe_ value. Of the two parameters *n* and *μ* (or *r*) utilised for describing superparamagnetism, the latter changes only slightly with *C*_Fe_ and *P*_Ar_, indicating that the former (*n*) is the key parameter affecting the magnetic properties. This is clearly observed from a comparison of the results of *M*_10_ as a function of *C*_Fe_ at various *P*_Ar_ values (Fig. 2) with those of *n* (Fig. 4(d)).

In order to explain the large change in the magnetic properties, further structural characterisations were performed. Plan-view HR-TEM images reveal a columnar structure, with its tendency stronger at a higher *P*_Ar_ value. An effort was then made to determine the relationship between the *n* value and the number density of columns (*n*_c_). Although it is difficult to estimate the *n*_c_ value accurately, mainly because of the fact that macro-columns usually consist of several micro-columns with blurred boundaries, the estimated *n*_c_ and *n* values are of a similar order. The *n*_c_ value of the annealed (57.9, 10) sample, whose column boundaries are clear, is 5.3 × 10^16^/cm^3^; this is similar to the *n* value (3.9 × 10^16^/cm^3^) extracted from Langevin fitting (see Supplementary Table S1). Despite this, the *n*_c_ value is unlikely to explain the observed magnetic properties. One reason for this unlikelihood is that only a slight change occurs in the columnar structure, including in the *n*_c_ value, upon annealing (Fig. 6(b) and (c) and Fig. 6(e) and (f)), which is in significant contrast to a considerably large change in the magnetic properties upon annealing (Fig. 3(a) and (b) and Fig. 4(d)). The remaining and the most probable factor for explaining the observed magnetic properties is the change in the degree of amorphization as a function of *P*_Ar_, because a more relaxed amorphous structure (or a smaller degree of amorphization) is expected at a smaller *P*_Ar_ value. XAFS was used to quantify the degree of amorphization; however, its results failed to provide solid evidence. One possible reason is the lack of reliable information on the Zr atom distribution. A large number of Bragg peaks originating from the Si/SiO_2_ substrate, some of which were very sharp, were observed near the Zr-K edge (17998 eV). An attempt was made to remove the Bragg peaks, but this resulted in a loss of absorption results and hence a decrease in the reliability in wave-vector (*k*) space and in the Fourier-transformed magnitude. It was not possible to examine the local structure of Zr atoms, which limited the analysis of the degree of amorphization in terms of both Fe and Zr atoms (see Supplementary Fig. S3). Another reason is the limitation of EXAFS for severely disordered systems^21^; the loss of low-*k* information during the Fourier transformation can limit the usefulness of EXAFS analysis, and in some cases, the σ^2^ value cannot describe the disorder owing to the limitation of the Debye–Waller approximation for disordered systems.

Despite this failure, the reason behind the large change in the magnetic properties is quite likely to be the change in the degree of amorphization as a function of *P*_Ar_. In the *P*_Ar_ range of 2–10 mTorr covered in this study, the difference in the degrees of amorphization is considered to be small. An indirect piece of evidence for this consideration is that the *M* values of the samples fabricated at *P*_Ar_ = 10 mTorr are extremely large, but after annealing at a low temperature of 150 °C for 30 min, they reduce significantly to a level similar to those of the as-deposited samples fabricated at *P*_Ar_ = 2 mTorr. This may indicate that the difference in the degrees of amorphization between the samples fabricated at *P*_Ar_ = 2 and 10 mTorr corresponds to the structural relaxation that occurs during the low-temperature annealing. Only a small amount of structural relaxation will occur during the low-temperature annealing; therefore, the difference in the degrees of amorphization is expected to be small in the considered *P*_Ar_ range. This indicates that even a small change in the degree in the amorphization can cause an extremely large change in the magnetic properties. It is known that the X-ray scattering method is one of the most advanced methods in quantifying the degree of amorphization. Even with the method, however, great difficulties arise in obtaining reliable results^22^,^23^. Given the great difficulty in quantifying the difference in amorphous structures, the magnetic properties of amorphous alloys can be utilised in evaluating the difference in amorphous microstructures. Indeed, this systematic study aimed at understanding the relationship between the degree of amorphization and the magnetic properties of amorphous Fe–Zr alloy thin films reveals a large *M* value at a high degree of amorphization over a wide composition range. Thus, if the relationship between the degree of amorphization and the magnetic properties is established for a certain system, these results can be utilised for inferring the amorphous structure from the magnetic properties. This approach can be an alternative way to overcoming the great difficulty in quantifying the degree of amorphization.

## Methods

Amorphous thin films with the Fe–Zr (200 nm)/Ta (5 nm) structure were deposited on wet-oxidised Si/SiO_2_ substrates by using a direct-current magnetron sputtering system. The base pressure of the sputtering chamber was 1 × 10^−7^ Torr, and the target-to-substrate distance was 60 mm. The sputtering power was 20 W, and the *P*_Ar_ value was varied to 2, 6, or 10 mTorr. The composition was controlled mainly by varying the number of Fe chips on an Fe_20_Zr_80_ (in at.%) alloy target. Some as-deposited samples were annealed in vacuum at 150 °C for 30 min. The magnetic properties were characterised using a vibrating sample magnetometer (VSM). The microstructural characterisations were performed by several methods, including XRD (using Cu Kα radiation), HR-TEM and STEM together with EDS elemental mapping, and XAFS measured at the Fe-K edge (7112 eV) in the fluorescence mode. The composition was analysed by an inductively coupled plasma method.

## Additional Information

**How to cite this article**: Kim, M. *et al*. Large variation of magnetic properties of amorphous Fe–Zr thin films with Ar pressure during sputtering. *Sci. Rep.* **7**, 41894; doi: 10.1038/srep41894 (2017).

**Publisher's note:** Springer Nature remains neutral with regard to jurisdictional claims in published maps and institutional affiliations.

## Supplementary Material

Supplementary Information
