# Supplementary material for: Large variation of magnetic properties of amorphous Fe–Zr thin films with Ar pressure during sputtering
Source: Sci Rep. 2017 Jan 31;7:41894. doi: 10.1038/srep41894 (PMC5282579; doi:10.1038/srep41894)
Supplement: Supplementary Information [file srep41894-s1.pdf]

Supplementary information

Large variation of magnetic properties of amorphous Fe–Zr thin films with Ar pressure during sputtering

Miri Kim[1], Nark-Eon Sung[2] & Sang Ho Lim[1,3,*]

[1]*Department of Nano Semiconductor Engineering, Korea University, Seoul 02841, Korea*

[2]*Energy and Environmental Research Team, Beamline Division, Pohang Accelerator Laboratory, Pohang 37673, Korea*

[3]*Department of Materials Science and Engineering, Korea University, Seoul 02841, Korea*

Elemental mapping results of as-deposited (60.0, 2) sample

The high-angle annular dark-field (HAADF) image and elemental mapping results for the as-deposited (60.0, 2) sample were obtained at a higher resolution than that of the results in Fig. 6; they are shown in Figs. S1(a)–(c). The columnar structure is clearly visible in the HAADF image in Fig. S1(a), as is the case with the result obtained at a lower resolution (Fig. 6(d)). A similar columnar structure is also visible in the Fe mapping result in Fig. S1(b), which is in significant contrast to the result obtained at a lower resolution (Fig. 6(g)), where no clear columnar structure is observed. The distribution of Zr is uniform, similar to that obtained at a lower resolution (Fig. 6(j)).

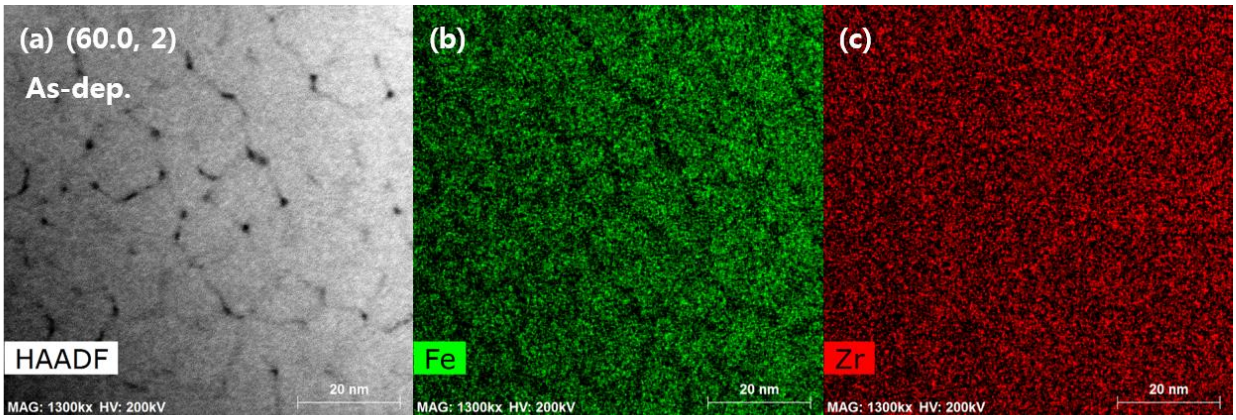

Figure S1. (a) HAADF image of as-deposited (60.0, 2) sample and elemental mapping results

for (b) Fe and (c) Zr.

X-ray absorption near-edge structure (XANES) spectra measured at Fe-K edge

XANES spectra at the Fe-K edge for the (41.8, 2), (40.5, 10), (60.0, 2), and (57.9, 10) samples in the as-deposited state (solid lines) and after annealing at 150°C (dashed lines) are shown in Fig. S2. Also shown in the figure are those obtained for reference samples of Fe, Fe_2O_3, Fe_3O_4, and FeO. All the samples show an absorption edge similar to Fe rather than to Fe oxides, indicating that that Fe atoms are in the metallic state even after the low-temperature annealing.

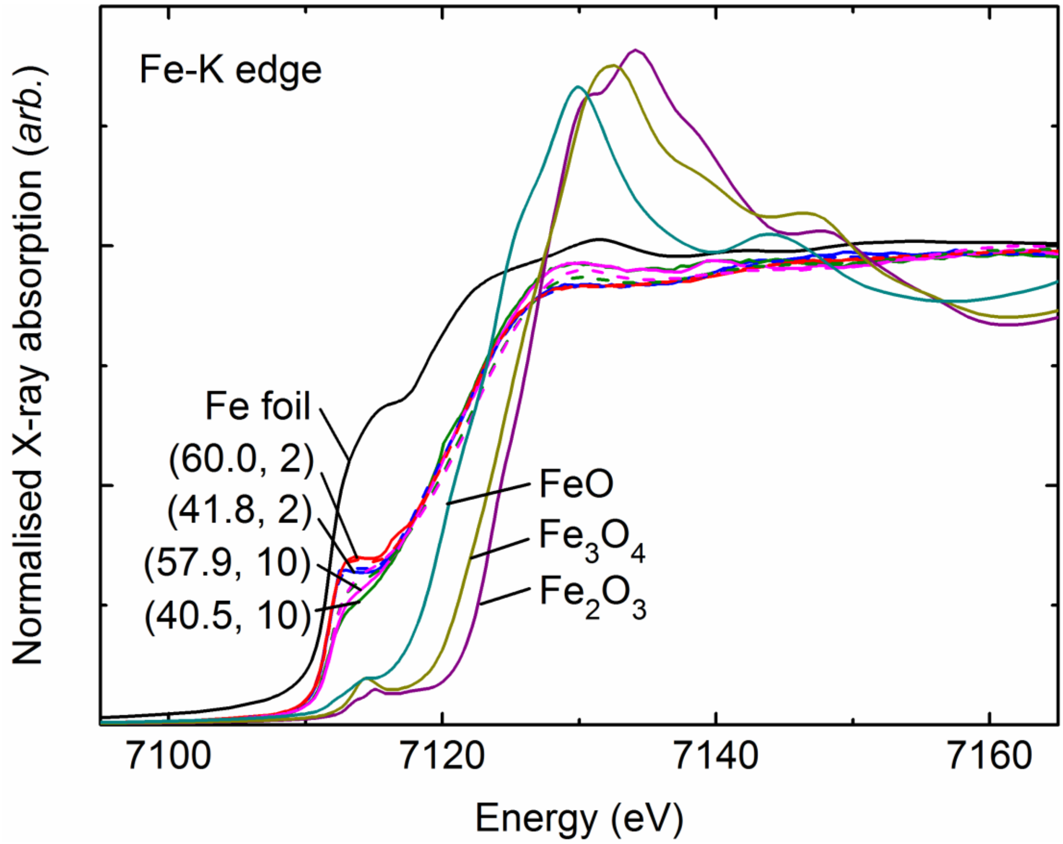

Figure S2. XANES spectra at Fe-K edge for (41.8, 2), (40.5, 10), (60.0, 2), and (57.9, 10) samples in as-deposited state (solid lines) and after annealing at 150°C (dashed lines), together with those for reference samples of Fe, Fe_2O_3, Fe_3O_4, and FeO.

Number density of columns from TEM images

Despite the difficulties in estimating the number density of columns (n_c) due mainly to blurred boundaries of micro-columns, the estimated n_c values are of an order similar to the

number density of superparamagnetic particles (n) extracted from Langevin fitting. However, no direct correlation appears to exist between n_c and n. For example, for the (57.9, 10) sample, the n_c values are similar both in the as-deposited and the annealed states, although the n values (and hence the magnetic properties) differ significantly.

Table S1. n values obtained from Langevin fitting and n_c values obtained from TEM images for as-deposited (60.0, 2) and (57.9, 10) samples and annealed (57.9, 10) sample.

Number density (/cm^3)	(60.0, 2) As-dep.	(57.9, 10) As-dep.	(57.9, 10) 150°C
n	1.6×10^{16}	8.9×10^{17}	3.9×10^{16}
n_c	1.4×10^{16}	6.4×10^{16}	5.3×10^{16}

X-ray absorption fine structure (XAFS) results measured at Zr-K edge

Figures S3(a)–(d) show XAFS results obtained at the Zr-K edge (17998 eV) for the (60.0, 2) and (57.9, 10) samples in the as-deposited and annealed states. The results reveal several Bragg peaks originating from the Si/SiO_2 substrate, some of which are sharp and strong; this makes it difficult to obtain results without Bragg peaks. At the Fe-K edge (7112 eV), as well, Bragg peaks attributed to the same Si/SiO_2 substrate are present, as can be seen in the inset of Fig. S3(b); however, these peaks are rather weak. In an effort to analyse the local structure of Zr more accurately, XAFS measurements were repeated, but no satisfactory results were obtained. An attempt was then made to remove the Bragg peaks, but this was achieved at the cost of a large loss of absorption results, thus resulting in a decrease in the reliability in wave-vector (k) space and in the Fourier-transformed magnitude. Because of this, the analysis of the XAFS results was focused only on the Fe atoms.

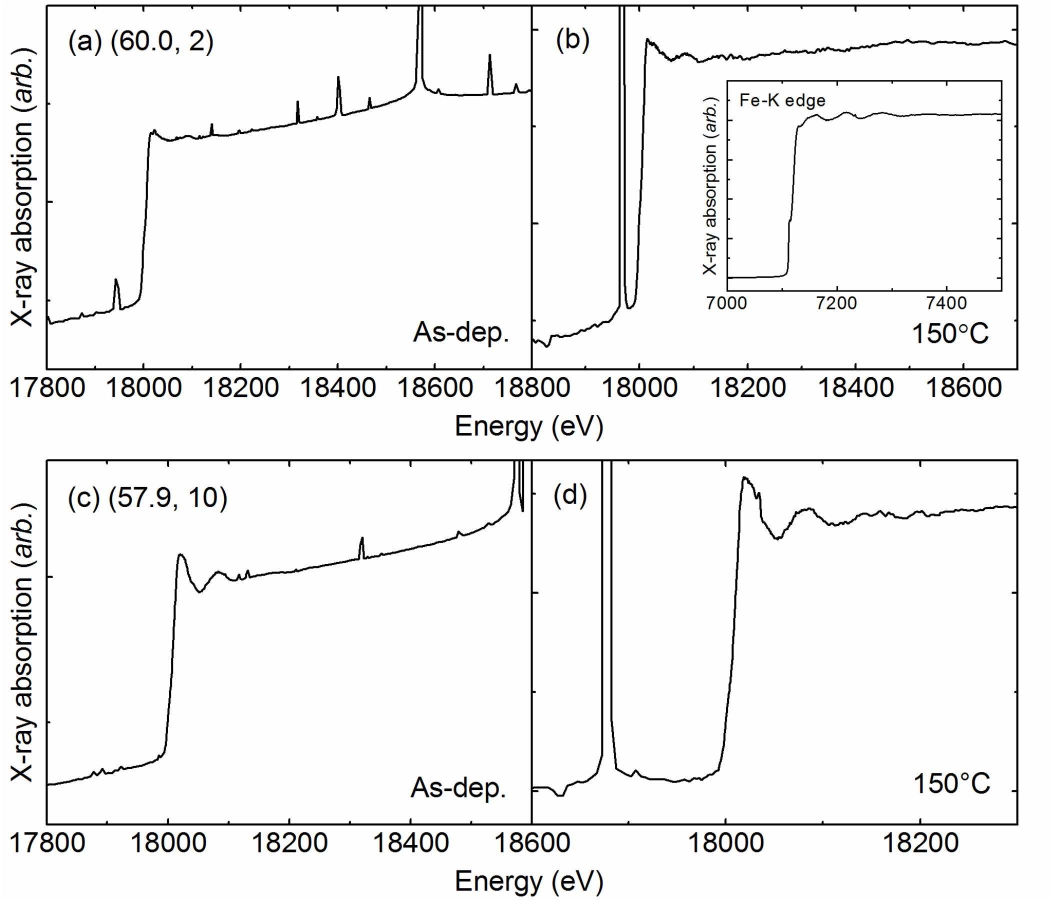

Figure S3. X-ray absorption curves at Zr-K edge for (60.0, 2) ((a) and (b)) and (57.9, 10) ((c) and (d)) samples in as-deposited and annealed states. The inset in (b) shows the X-ray absorption curve at the Fe-K edge for the (60.0, 2) sample in the annealed state.
